# Analysis of Home-Based Rehabilitation Awareness, Needs and Preferred Components of Elderly Patients with Hip Fracture Surgery in South Korea

**DOI:** 10.3390/ijerph18147632

**Published:** 2021-07-18

**Authors:** Haneul Lee, Seon-Heui Lee

**Affiliations:** 1 Department of Physical Therapy, College of Health Science, Gachon University, Incheon 21936, Korea; leehaneul84@gachon.ac.kr; 2 Department of Nursing, College of Nursing, Gachon University, Incheon 21936, Korea

**Keywords:** older, hip fracture, home-based rehabilitation, multicomponent, awareness

## Abstract

The importance and necessity of home-based rehabilitation with professional and systematic interventions should be considered since home-based rehabilitation has been institutionalized as it is more feasible, cost effective, and even safer than in-hospital rehabilitation in most countries—though not in South Korea. In addition, the need for home-based rehabilitation is increasing due to the increasing number of hip fracture patients and limited capacity of acute hospital rehabilitation. Therefore, the purpose of this study was to investigate the awareness, needs, and preferred components of home-based rehabilitation services after discharge for elderly patients with hip fracture surgery in South Korea. A survey of 98 elderly patients who recently underwent hip fracture surgery was performed using a questionnaire. More than 75% of patients agreed on the need for home-based rehabilitation, even though most had never heard of it. The reason for the need for home-based rehabilitation was that it is possible to receive continuously ongoing treatment (53.0%), and it alleviates the inconvenience of visiting hospitals (27.7%). In addition to this, about 15.7% of patients responded that they could achieve mental comfort. In other words, patients can recover in an emotionally stable environment without the psychological anxiety they might experience in hospital. Thus, in order to maximize the effectiveness of home-based rehabilitation and provide comprehensive guidance including exercise, education, motivational support, and environmental modification, to patients undergoing hip fracture surgery, the component of the rehabilitation program must be developed based upon rehabilitation experts’ knowledge and patients’ value. Additionally, corresponding policies should be established.

## 1. Introduction

Hip fracture is a serious injury that requires immediate medical care, especially among older people. Most hip fractures occur in adults aged 65 years and older [1]. The incidence of hip fractures rises with age, and nearly doubles after the age of 50. In addition, hip fracture is more common in women because women have a higher rate of osteoporosis [1,2]. By 2050, the annual number of hip fractures is expected to rise to 6.26 million, with a huge increase among the elderly population, which could be a potential global economic burden [2,3]. The number of hip fractures is expected to continuously increase with a direct medical cost of over USD 10 billion each year by 2050 due to the growing aging population in Asian counties [3]. According to recent epidemiological evidence on hip fracture in South Korea, the number of hip fracture patients among those aged 50 and older has been rising every year [4,5]. South Korea became an aging society with an aging rate of 7.2% in 2000; this figure increased to 14.3% in 2018, and the aging rate is expected to continue to rise to 42.5% by 2065, the world’s highest [6]. The incidence of hip fractures among the elderly in South Korea has also expanded as the national population continues to age; the number of hip fractures has increased by 2025, which will lead to a socioeconomic burden [7].

According to the World Health Organization report, among the major musculoskeletal conditions’ burden, hip fracture is the most damaging fracture, being associated with 20% mortality and 50% permanent loss in function [8]. The 1-year mortality rate after hip fracture is approximately 22%, and the first year after a hip fracture is the most critical period [9]. Pain, decreased ROM, and weak muscle strength are associated with poor balance and gait speed, which affect mobility [10,11]. Although surgical treatment can shorten one’s hospital stay and the duration of pain, as well as improve patient function associated with hip fracture in elderly patients, long-term rehabilitation is required for functional independence [12]. The length of hospital stay has shortened due to the shortage of hospital beds and rising medical expenses, which has led to interest in home-based rehabilitation for ongoing rehabilitation care after discharge [13]. Furthermore, home-based rehabilitation for various ailments has a positive effect on improving patients’ physical and mental functions [14,15,16,17].

Accelerated discharge from hospital and home-based rehabilitation has been institutionalized in most countries, as it is more feasible, cost effective, and safer than in-hospital rehabilitation, but not in certain countries, including South Korea [18,19]. In South Korea, home-based rehabilitation provides only indirect care services such as counseling on health issues, basic nursing care, and health education for very limited low-income population from some public health centers and local welfare service centers for the disabled [20]. In addition, the need for home-based rehabilitation is increasing due to the increasing number of hip fracture patients and the limited capacity in acute hospital rehabilitation. Thus, the importance and necessity of home-based rehabilitation with professional and systematic interventions should be considered [14,15,16,17].

**Figure 1:**
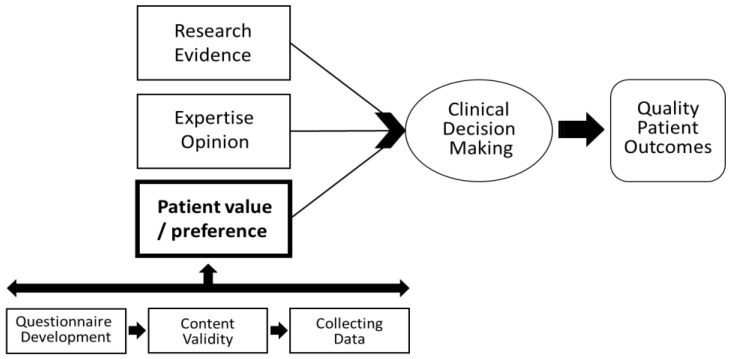
Research framework [21].

To establish home rehabilitation system which can improve the clinical outcome for older people suffering from hip fracture, evidence-based practice (EBP) would be an option, which is a good life-long problem-solving approach that integrates the best evidence of studies concerned, experts’ opinion and patients’ preference (Figure 1) [21]. A lot of evidence already supports that home-based rehabilitation has a positive effect on improving patients’ physical and mental functions and many experts assured that the importance of home-rehabilitation should be highlighted [14,15,16]. However, there is still a lack of studies to infer patient’s value on the component of home-based rehabilitation for older people with hip fracture surgery. Therefore, the purpose of this study was to investigate the awareness, needs, and preferred components of home-based rehabilitation services based on developed questionnaires by expertise after discharge for elderly patients with hip fracture surgery in South Korea.

## 2. Materials and Methods

The study was conducted according to the guidelines of the Declaration of Helsinki, and approved by the Gachon University Institutional Review Board (1044396-201911-HR-209-02). The purpose of this study and protection of the privacy of participants were fully explained before the survey was conducted. Once the participant agreed to participate in the survey, they signed their informed consent before beginning of the survey.

### 2.1. Research Framework

For the evidence-based practice (EBP) that integrates the best evidence of studies concerned, experts’ opinion and patients’ preference, this study was to investigate the awareness, needs, and preferred components of home-based rehabilitation services based on the developed questionnaire by expertise.

### 2.2. Participants

This study included patients in the study if they met the following inclusion criteria: (1) 65 years or older; (2) admitted to a medical center for a traumatic hip fracture; and (3) underwent surgery to treat hip fracture within 4 months. We excluded people who were unable to perform the survey, such as those who with neurologic disorders who could not perform ADL before the fracture, cognitive impairment or dementia (Mini-Mental State Examination score < 24) or who were unable to communicate.

We distributed 118 questionnaires to 17 medical centers in South Korea and 100 questionnaires returned, and among them, two questionnaires did not meet the inclusion criteria. We administered a survey to 98 elderly patients post-hip fracture surgery from May to August 2020.

### 2.3. Preliminary Evaluation Questionnaire Development

We developed the questionnaire used in this study to examine the home-based rehabilitation awareness and needs of patients undergoing hip fracture surgery. A survey entitled, “Questionnaire on the awareness and needs regarding home-based rehabilitation programs for elderly patients after hip fracture surgery in South Korea” was developed. To elaborate the questionnaire, we prepared a draft in accordance with the circumstances of South Korea after several meetings between researchers with reference to previous survey methods [19,22]. Then, a panel of experts investigated the validity of the draft to determine whether it included appropriate components for the study’s purpose and if it was appropriate for patients. The expert panel consisted of two physiatrists, two physical therapists, two nurses, two professors in the physical therapy department, and two professors in the nursing department; two repeated surveys were conducted. The content surveyed included the participants’ general characteristics, their awareness and needs surrounding home-based rehabilitation, and the components of home-based rehabilitation programs for patients undergoing hip fracture surgery.

### 2.4. Quantification of Content Validity

The content validity of the questionnaire was determined by the panel of experts who conducted the verification of the preliminary evaluation questionnaire. Item-level content validity index (I-CVI) was calculated [23] using a 4-point scale regarding the importance and feasibility of the components of home-based rehabilitation programs for elderly patients following hip fracture surgery. Then, scores of less than 0.8 of I-CVI from the list were excluded. Furthermore, we excluded “aerobic exercise” based on experts’ belief that “aerobic exercise” and “endurance exercise” are used in the same sense. Therefore, we excluded “aerobic exercise,” “therapeutic modalities,” and “manual therapy” from the list. In addition, we merged “exercise” and “rehabilitation therapy” under the duty category into “therapeutic exercise” to avoid redundant meanings. We added “education for fall prevention” based on experts’ opinions.

The final questionnaire involved: (1) the respondents’ basic information; (2) awareness and needs of home-based rehabilitation services; and (3) the importance of the components of home-based rehabilitation services. The questions on “basic information” included gender, age, education, type of residence, primary caregiver, economic status, experience of falling, and walking status before fracture. For the “awareness and needs of home-based rehabilitation services,” we included the elements of awareness of home-based rehabilitation services, the need for such a service, use of this service, and preferred frequency, duration and cost. Questions related to “the importance of the components of home-based rehabilitation services” were rated using a 5-point Likert scale (Supplementary Materials Figure S1).

### 2.5. Collecting Data

We conducted the survey between May and August 2020. We distributed the questionnaire to patients who had undergone surgery after hip fracture. The study’s purpose was described on the first page of the questionnaire, and the questionnaire collected during the survey period was used for analysis.

### 2.6. Statistical Analysis

The analysis of this study was largely carried out in two aspects: home-based rehabilitation awareness and needs of patients undergoing hip fracture surgery, and the importance of the components of home-based rehabilitation services. Each question contains a variety of other questions, depending on its characteristics, such as a “5-point Likert scale question,” “a single choice item,” “a multiple choice item,” and “yes/no choice item.” For data analysis, frequency analysis was performed according to each item’s characteristics, and the data were calculated as means and standard deviations (SD) for quantitative variables, as well as for the frequency and percentage for categorical variables using IBM SPSS Statistics for Windows (Version 23.0, IBM Corp., Armonk, NY, USA). The level of significance was set at α = 0.05.

## 3. Results

### 3.1. Patients’ Awareness and Needs for Home-Based Rehabilitation Programs: The Survey

Total of 98 questionnaires were included for statistical analysis. The characteristics of the participants who participated in the survey are shown in Table 1. Regarding age brackets, 35 (39.3%) participants were in their 60s, followed by 32 (36.0%) in their 70s, 19 (21.3%) in their 80s, and 3 (3.4%) in their 90s or above. The participants consisted of 63 women (64.3%) and 35 men (35.7%). The median days after the hip surgery of patients was 30 days, with an interquartile range (IQR) of 7 to 72. Sixty-five patients (66.3%) were able to walk independently without any assistive device, and 38 patients (38.8%) experienced falling before their fracture.

### 3.2. Patients’ Awareness and Needs of Home-Based Rehabilitation Programs

#### 3.2.1. Experience of Hearing about Home-Based Rehabilitation Services

Table 2 shows whether respondents who participated in the survey had heard of home-based rehabilitation program services. Twenty-six (26.5%) responded “yes,” while 72 (73.5%) answered “no.”. It was found that most patients had never heard of home-based rehabilitation services.

#### 3.2.2. Whether Home-Based Rehabilitation Services Are Needed

The participants were asked to respond on a 5-point Likert scale to determine whether they needed home-based rehabilitation services, as patients with either disabilities or limited ADL. Of the total respondents, 75.6% (strongly agree and agree) agreed for the need for home-based rehabilitation services and only 12.2% of patients disagreed.

#### 3.2.3. Whether to Participate in Home-Based Rehabilitation Services

Regarding the question “Would you like to participate in the service when the home-based rehabilitation service is established?” Eighty-three (84.7%) respondents said “yes” while 13 (13.30%) said “no.” We found that most patients were willing to participate in home-based rehabilitation services when they are launched in South Korea.

As for follow-up questions, for patients who responded that they would participate in home-based rehabilitation services, 44 (53.0%) mentioned that “I will likely receive ongoing treatment, even after I am discharged.” Moreover, 23 (27.7%) subjects expressed that there would likely be less of a burden when visiting the hospital, 13 (15.7%) said “I am likely to be mentally comfortable,” and 3 (3.6) asserted “there will likely be less of a burden regarding time.”

On the other hand, for patients who said they would not participate in home-based rehabilitation services, eight (61.5%) said they “prefer continued hospitalization or outpatient rehabilitation,” four (30.8%) pointed to a lack of reliability of treatment tools or visiting therapists, and one (7.7%) said, “Medical expenses will likely be higher.”

#### 3.2.4. Preferred Number of Home-Based Rehabilitation Treatment Visits, Duration and Cost

Most respondents—40 people (40.8%)—thought that a proper number of home-based rehabilitation treatment visits was “three times a week”, followed by 23 people (23.5%) who thought “twice a week”. The most appropriate time for treatment was 1 h for 51 patients (52.0%), followed by 30 min for 22 patients (22.4%), and 45 min for 15 patients (15.4%). Among the respondents, 45 people (45.9%) said that the appropriate cost of home-based rehabilitation was “maintaining the current level” (current level: KRW 8170 (USD 7.3) per hour) and 26 people (26.5%) said it should be “less than KRW 5000 (USD 4.4)” (Table 3).

### 3.3. The Importance of the Components of Home-Based Rehabilitation Programs

Table 4 shows the importance of the components of home-based rehabilitation programs for patients with hip fracture surgery. Questions were rated using a 5-point Likert scale. As for the patient evaluation score for the importance of the components of home-based rehabilitation programs, “ambulation exercises” were considered the most critical (4.33), followed by “ROM exercises” (4.28) and “strengthening exercises” (4.19), which were also selected as a component of high importance. On the other hand, “pressure ulcer management” (3.37), the “ability to handle medication” (3.38), and “vitamin supply counseling” (3.39) were selected as components that were considered less important.

## 4. Discussion

With this study, we aimed to examine the awareness, needs, and preferred components of home-based rehabilitation services for elderly patients having undergone hip fracture surgery after discharge. This survey indicates that establishing a home-based rehabilitation service is necessary for patients having undergone hip fracture surgery.

### 4.1. Awareness and Needs of Home-Based Rehabilitation Services

Although only 26.5% of patients said they knew about home-based rehabilitation, more than 75% agreed on the need for it. Our result supports the recent survey of necessity on home visiting physical therapy service in Korea. Kim showed that about 75% patients agreed on the need of home-based rehabilitation [20]. Similarly, 67.2% of caregivers of pediatric patients were not aware of home-based rehabilitation [24]. Previous research showed that even approximately 50% of physical therapists were not aware that home-based rehabilitation was currently not being implemented in Korea [25]. On the other hand, Lester and Gibson showed the overall awareness of home rehabilitation was favorable even though this study was conducted almost 20 years ago [26]. Additionally, it is very similar to our results that most of respondents had not heard of the program, but said that if the program was implemented, they would like to participate in it [20,24,25]. These studies were conducted on a different group of patients or a caregiver who had received physical therapy services at least once rather than in a particular patient group, but the results were almost identical to our paper [20,24,25]. This shows that most patients want home-based rehabilitation to be implemented.

### 4.2. Preference to Participate in Home-Based Rehabilitation Services

In this study, for patients who responded for participating in the home-based rehabilitation service, 44 (53.0%) respondents answered “it is likely to have continuous treatment even after discharge”, and 23 (27.7%) answered “it’s likely to be less burden visiting hospital”. This is similar to previous studies suggesting that home-based rehabilitation can continue treatment even after discharge and reduce discomfort in visiting the hospitals [20,24,25]. In addition to this, about 15.7% of patients responded that it would help achieve mentally (psychological) comfort. In other words, patients can recover in an emotionally stable environment without the psychological anxiety they might experience in hospital. Thus, home-based rehabilitation is expected to play an important role in reducing the physical as well as mental burden of patients who need long-term care.

As a result, the lack of awareness among patients appears, and the need for active publicity and increased awareness of home-based rehabilitation services are generally high, indicating that the medical system should implement home-based rehabilitation services as soon as possible.

### 4.3. The Importance of the Components of Home-Based Rehabilitation Programs

The components of rehabilitation programs are important for effective rehabilitation. Since rehabilitation is conducted at home, modifications to the home environment, education, nutrition, and motivational counseling should be included in rehabilitation programs to improve physical functions and emotional status [13,27,28]. Lee and Yu found that multifactorial interventions with environmental modifications were significantly more effective in preventing falls than usual care, but not in multifactorial interventions without environmental modifications [28]. As far as the importance of implementing a home-based rehabilitation service is concerned, the components of the rehabilitation program should be well organized.

Evidence-based practice requires integrating the best research evidence with clinical expert judgments, as well as the patient’s values and expectations [29]. Thus, we investigated the needs and importance of the components of home-based rehabilitation programs from patients’ perspectives based on experts’ consensus. A questionnaire developed by experts was administered regarding the importance of program components for hospitalized patients undergoing hip fracture surgery. In the experts’ view, the most important category of the program was “exercise”, such as ROM exercises, ambulation, strengthening, and balance. The least important components were therapeutic modalities, manual therapy, and nutrition counseling. Therapeutic modalities and manual therapy are considered less feasible. Since the CVI of importance or feasibility is less than 0.8, we excluded therapeutic modalities and manual therapy from our home-based rehabilitation program for elderly patients with hip fracture surgery. This may be due to the fact that home-based rehabilitation is often conducted in limited space and with limited instruments. Patients’ score for the importance of program components was somewhat lower than that of the expert group, but the result is consistent in terms of the “exercise” category being the most important.

As for the patient evaluation score, ambulation exercises were considered the most important, followed by ROM and strengthening exercises. Patients considered pressure ulcer management as the least critical, while the expert group considered this item to be quite vital. Patients and experts disagreed on the “education” category except for positioning education, and modifications of the home environment. This is probably because patients focused more on treatments with immediate effects, while experts thought of integrated treatment from a broader perspective. However, “education” is a crucial element of rehabilitation programs, and a lot of evidence has revealed its effectives [13,30,31]. Education, along with emotional (motivational) support, has a significant impact on adherence to home exercises, which leads to better treatment outcomes, so this aspect should not be removed from rehabilitation programs [32].

### 4.4. Advantages of Home-Based Rehabilitation Program for Elderly Patients with Hip Fracture Surgery

In addition, legal and institutional support is required which enables patients to continuously receive rehabilitation care in the community after getting discharge from the hospital in order to activate the early discharge of patients who undergo surgery after a hip fracture, [33]. They did not provide sufficient rehabilitation programs after discharge from the hospital, resulting in many problems such as joint contractures, limited daily activities, functional status, and quality of life (QoL). Many studies have revealed the positive effects of home-based rehabilitation services on functional recovery and reinforcement to enable daily activities in order to prevent disability, shorten hospital stay, offer emotional stability, and reduce medical costs—which is comparable to in-hospital rehabilitation [17,27]. In terms of low-cost, convenience, and diverse home-based rehabilitation services, community-oriented, home-based rehabilitation services are attracting attention in most countries, but they have been primarily established in Western countries [34]. Moreover, when compared with in-hospital rehabilitation, physical function outcome after the home-based rehabilitation for elderly patients with hip fracture surgery was comparable to inpatient rehabilitation [13,35,36].

It is expected that the economic burden of medical expenses and time consumed can be reduced by minimizing unnecessary hospital use and transfer to other facilities, and that home-based rehabilitation will be able to meet the needs of hip fracture patients who need rehabilitation after discharge [37]. In particular, the provision of home-based rehabilitation to elderly disabled patients with hip fractures may enable independent daily activities as much as possible, which can lead to various economic and social benefits.

### 4.5. Limitations

This study has the following limitations. Even though our selection criteria for participation were very strict, we did not consider the clinical condition (e.g., level of disability, complications or comorbidity) of participants. Since the levels of disability, assistance, or complications after hip fracture surgery greatly vary according to the type of fracture and the type of surgical intervention, the respondent’s thoughts could be interfered with by these their conditions. Additionally, further study should explore the home-rehabilitation service from the caregiver’s point of view of home-based rehabilitation service, since elderly patients with hip fracture surgery need a lot of care,

## 5. Conclusions

In summary, about 75% elderly patients with hip fracture surgery agreed on the need for home-based rehabilitation and wanted to participated. The component of rehabilitation program must be developed based on rehabilitation experts’ knowledge and patients’ values in order to maximize the effectiveness of home-based rehabilitation and provide comprehensive guidance to patients undergoing hip fracture surgery. Additionally, corresponding policies should be established to actively pursue community health care and private rehabilitation institutions. Hence, home-based rehabilitation will not only meet the needs of elderly patients who undergo hip fracture surgery, but can also provide continuous rehabilitation, even after discharge, in an emotionally stable environment.

## Figures and Tables

**Table 1:** General characteristics of the participants (*n* = 98).

Characteristics	
Age (year), mean ± SD	Total
65–69 years, *n* (%)	73.1 ± 8.7
70–79 years	35 (39.3)
80–89 years	32 (36.0)
90 years or older	19 (21.3)
Gender (women), *n* (%)	3 (3.4)
Education, *n* (%)	63 (64.3)
No education or elementary school	
Middle school	39 (39.8)
High school	16 (16.3)
Undergraduate or graduate school	32 (32.6)
Living status, *n* (%)	11 (11.2)
Alone	
With partner	23 (23.5)
With partner and son or daughter	34 (34.7)
With siblings	12(12.2)
Nursing home	16 (16.3)
Economic status, *n* (%)	13 (13.3)
Low	
Middle	44 (44.9)
High	25 (25.5)
Caregiver	29 (29.6)
Partner	
Son or daughter	31 (31.6)
Cousin	14 (14.3)
Professional caregiver	1 (1.0)
Days after hip surgery (days), median (IQR)	52 (56.0)
Walking independently before fracture, *n* (%)	30 (7–72)

**Table 2:** Patients’ awareness and needs of home-based rehabilitation programs (*n* = 98).

Items	*n* (%)
Have you heard of home-based rehabilitation?	
Yes	26 (26.5)
No	72 (73.5)
Necessity of home-based rehabilitation	
Strongly agree and agree	74 (75.6)
Strongly disagree and disagree	12 (12.2)
Neither agree nor disagree	12(12.3)
Will you participate in home-based rehabilitation?	
Yes	83 (84.7)
No	13 (13.3)
The reasons for participating in home-based rehabilitation	
Mentally comfortable	13 (15.7)
A lighter burden in terms of time	3 (3.6)
Ongoing treatment even after discharge	44 (53.0)
Less of a burden in the hospital	23 (27.7)
The reason for not participating in home-based rehabilitation	
Prefer continued hospitalization or outpatient rehabilitation	8 (61.5)
Lack of reliability of intervention tools or visiting therapist	4 (30.8)
More medical expenses	1 (7.7)

**Table 3:** Preferred number of home-based rehabilitation treatment visits, duration and cost (*n* = 98).

Items	*n* (%)
Number of visits	
Once a week	12 (12.2)
Twice a week	23 (23.5)
Three times a week	40 (40.8)
Four times a week	6 (6.1)
Five times a week	17 (17.3)
Duration of one visit	
30 min	22 (22.4)
45 min	15 (15.3)
60 min	51 (52.1)
90 min	6 (6.1)
120 min	4 (4.1)
Medical cost of one visit	
Below KRW 5000 (USD 4.4)	26 (26.5)
Current level (8170 (USD 7.3) home visiting nursing service)	45 (45.9)
KRW 10,000 (USD 8.9)	19 (19.4)
KRW 15,000 (USD 13.3)	4 (4.1)
over 20,000 (USD 17.7)	4 (4.1)

**Table 4:** The importance of the components of home-based rehabilitation programs for patients undergoing hip fracture surgery (*n* = 98).

Duty	Task	Importance
Therapeutic exercises	Strengthening exercises	4.19
	Endurance exercises	3.86
	Range of Motion exercises	4.28
	Breathing exercise	3.31
	Balance exercise	4.14
	Ambulation exercise	4.33
	Activities of daily living training	3.94
Education	Positioning education	4.15
	Fall prevention	4.06
	Training in the use of assistive devices	3.86
	Ability to handle medication	3.38
	Caregiver education	3.69
Pressure ulcer	Pressure ulcer management	3.37
Nutrition	Provided nutrition advice	3.45
	Vitamin supply counselling	3.39
Environmental	Modifications of the home environment	3.83

## Data Availability

No new data were created or analyzed in this study. Data sharing is not applicable to this article.

## Supplementary Materials

The following are available online at https://www.mdpi.com/article/10.3390/ijerph18147632/s1. Figure S1: Developed questionnaire on home-based rehabilitation awareness, needs, and preferred components of elderly patients with hip fracture surgery in South Korea.
