# Supplementary material for: Analysis of Home-Based Rehabilitation Awareness, Needs and Preferred Components of Elderly Patients with Hip Fracture Surgery in South Korea
Source: Int J Environ Res Public Health. 2021 Jul 18;18(14):7632. doi: 10.3390/ijerph18147632 (PMC8306015; doi:10.3390/ijerph18147632)
Supplement: Supplementary file 1 [file ijerph-18-07632-s001.zip › ijerph-1263687-SI.pdf]

Questionnaire on Home-Based Rehabilitation Awareness, Needs and Preferred Components of Elderly Patients with Hip Fracture Surgery in South Korea

[Consent]
Welcome. Thank you for taking part in this study. This study examines awareness, needs and preferred components of home-based rehabilitation for elderly patients with hip fracture surgery. You may be asked to respond to some questionnaires related to your personal information. There is no right or wrong answer, so please give us your honest thoughts about the home-based rehabilitation.

This study should take **about 15 minutes to complete**, but the exact time depends on your individual performance.

Your participation is voluntary. **Your responses will remain confidential and will only be treated in an aggregate fashion**. The results of the study may be published. You have right to withdraw from the study at any time. If you agree to participate the study, please sign the consent form. Consent is implied with a submission of the survey. Again, we appreciate your participation.

Section I. Demographics

1. What is your age? (   )

2. What is your gender?

① Male ② Female

3. What is the highest level of education?

① No formal education ② Elementary school ③ Middle school

④ High school ⑤ College/University ⑥ Graduate school

4.Which of the following living status best describes your household?

① Live alone ② Live with spouse only ③ Live with spouse and son or daughter

④ Live with siblings ⑤ Nursing home ⑥ Live with other unrelated

5. What is your household income?

① No income ② under ₩ 1,000,000 ($ 885) ③ ₩ 1-2,000,000 ($ 885 – 1,770)

④ ₩ 2-3,000,000 ($ 1,770 – 2,655) ⑤ over ₩ 3,000,000 ($ 2,655)

6. Who is responsible for your medical expenses?

① Myself ② Partner/Spouse ③ Son and daughters

④ Siblings or relatives ⑤ Insurance company ⑦ etc (   )

7. Who is your primary caregiver?

① Partner/Spouse ② Son or daughters ③ Relatives

④ Parents ⑤ Professional caregiver ⑧ etc ( )

8. Are there any other disease that have been diagnosed besides hip fractures?

9. How many days have passed since the hip fracture? (days after hip fracture)

10. How was the ambulation condition before the hip fracture?

① Walking independently without assistive devices

② Walking independently with assistive devices (cane or walker)

③ Walking with assists of one more person

④ Dependent in ambulation

11. Have you experienced falling in the past year?

① Yes (→ go to Q13) ② No (→ go to Q14)

12. How many falls have you experience in the past year? ( )

Section II. Awareness and needs of home-based rehabilitation services

> ※ Definition of **"home-based rehabilitation"**
>
> Providing rehabilitation services to patients with health-related problems by visiting their homes for rehabilitation program including therapeutic exercise, education, and environmental modifications etc.

13. Have you heard of home-based rehabilitation?

① Yes ② No

14. Do you think home-based rehabilitation services are necessary for elderly patients undergo hip fracture surgery?

① Strongly Agree ② Agree ③ Neither Agree/Disagree

④ Disagree ⑤ Strongly Disagree

15. Will you participate in home-based rehabilitation once it is established?

① Yes (→ go to Q16) ② No (→ go to Q17)

16. ("Yes" for Q15) What is the reason for participating the service?

① Mentally comfortable

② A lighter burden in terms of time

③ Ongoing treatment even after discharge

④ Less of a burden in the hospital

⑤ etc ( )

17. ("No" for Q15) What is the reason for not participating the service?

① More medical expenses

② Prefer continued hospitalization or outpatient rehabilitation

③ Do not feel comfortable visiting strangers

④ Lack of reliability of intervention tools or visiting therapist

⑤ etc ( )

18. How many visits do you think is appropriate for home-based rehabilitation service?

① Once a week ② Twice a week ③ Three times a week

④ Four times a week ⑤ Five times a week

19. How many hours do you think is appropriate for home-based rehabilitation service?

① 30 minutes ② 43 minutes ③ 60 minutes

④ 90 minutes ⑤ 120 minutes

20. How much do you think is appropriate for home-based rehabilitation service? (current level ₩ 8,170 per hour)

① Below ₩ 5,000 ($ 4.4) ② Current level (₩ 8,170 / $ 7.3) ③ ₩ 10,000 ($ 8.9)

④ ₩ 15,000 ($ 13.3) ⑤ Over ₩ 20,000 ($ 17.7)

Section III. The components of home-based rehabilitation program

■ Please rate the most importance of each of the following components of home-based rehabilitation program patients undergoing hip fracture surgery

Duty	Task	Importance				
		Not at all important	Low Importance	Neutral	Important	Very important
		1	2	3	4	5
Therapeutic exercises	21-1. Strengthening exercises					
	21-2. Endurance exercises					
	21-3. Range of Motion exercises					
	21-4. Breathing exercise					
	21-5. Balance exercise					
	21-6. Ambulation exercise					
	21-7. Activities of daily living training*					
Education	22-1. Positioning education					
	22-2. Fall prevention					
	22-3. Training in the use of assistive devices*					
	22-4. Ability to handle medication					
	22-5. Caregiver education					
Pressure ulcer	23-1. Pressure ulcer management					
Nutrition	24-1. Provided nutrition advice					
	24-2. Vitamin supply counselling					
Environmental	25-1. Environmental modification*					
Comments						

※ Activities of daily living training includes personal hygiene, bathing, feeding, dressing, bowel & bladder control, etc.

※ assistive devices refer to overall aids used by patients such as cane, walker, wheelchair, etc.

※ Environmental modification includes improvements (structural changes) in the space in which the patients reside.

- Thank you for your participating -

Figure S1. Developed questionnaire on home-based rehabilitation awareness, needs, and preferred components of elderly patients with hip fracture surgery in South Korea
